# Risk‐of‐falling related outcomes improved in community‐dwelling older adults after a 6-week sideways walking intervention: a feasibility and pilot study

**DOI:** 10.1186/s12877-021-02010-6

**Published:** 2021-01-14

**Authors:** Andreas Skiadopoulos, Nick Stergiou

**Affiliations:** 1 Department of Biomechanics and Center for Research in Human Movement Variability, University of Nebraska at Omaha, Biomechanics Research Building 214, 6160 University Drive South, 68182-0860 NE Omaha, USA; 2 College of Public Health, University of Nebraska Medical Center, 68198-4355 Omaha, NE USA

**Keywords:** Aging, Balance, Fear of falling, Gait, Stability, Variability, Lateral stepping

## Abstract

**Background:**

Aging increases fall risk and alters gait mechanics and control. Our previous work has identified sideways walking as a potential training regimen to decrease fall risk by improving frontal plane control in older adults’ gait. The purposes of this pilot study were to test the feasibility of sideways walking as an exercise intervention and to explore its preliminary effects on risk-of-falling related outcomes.

**Methods:**

We conducted a 6-week single-arm intervention pilot study. Participants were community-dwelling older adults ≥ 65 years old with walking ability. Key exclusion criteria were neuromusculoskeletal and cardiovascular disorders that affect gait. Because initial recruitment rate through University of Nebraska at Omaha and Omaha community was slower than expected (3 participants∙week^− 1^), we expanded the recruitment pool through the Mind & Brain Health Labs registry of the University of Nebraska Medical Center. Individualized sideways walking intervention carried out under close supervision in a 200 m indoor walking track (3 days∙week^− 1^). Recruitment and retention capability, safety, and fidelity of intervention delivery were recorded. We also collected (open-label) walking speed, gait variability, self-reported and performance-based functional measures to assess participants’ risk-of-falling at baseline and post-intervention: immediate, and 6 weeks after the completion of the intervention.

**Results:**

Over a 7-month period, 42 individuals expressed interest, 21 assessed for eligibility (21/42), and 15 consented to participate (15/21). Most of the potential participants were reluctant to commit to a 6-week intervention. Desired recruitment rate was achieved after revising the recruitment strategy. One participant dropped out (1/15). Remaining participants demonstrated excellent adherence to the protocol. Participants improved on most outcomes and the effects remained at follow-up. No serious adverse events were recorded during the intervention.

**Conclusions:**

Our 6-week sideways walking training was feasible to deliver and demonstrated strong potential as an exercise intervention to improve risk-of-falling outcomes in community-dwelling older adults. In a future trial, alternative clinical tools should be considered to minimize the presence of ceiling/floor effects. A future large trial is needed to confirm sideways walking as a fall prevention intervention.

**Trial registration:**

ClinicalTrials.gov identifier: NCT04505527. Retrospectively registered 10 August 2020.

## Background

Falls is the leading cause of injury among older adults aged 65 years and above [1, 2]. Falling threatens functional independence, increase disability and mortality, and financially burdens the patients and their caregivers [3–6]. Besides the health concern, falls result in substantial medical expenditures for the healthcare system, which is expected to exceed $100 billion by 2030 in the US [7], and €45 billion by 2050 in the EU [2]. Fall prevention interventions have been a major focus of research in recent years [8–13]. It is a particularly pressing topic due to the increase of the aging population and the growing awareness of the societal burdens resulting from falls [8].

### Frontal plane gait stability

The highest proportion of falls in older adults occurs during level walking [14–17]. Maintaining walking balance in older adults is a requirement for avoiding falls. However, with advancing aging, declines in sensorimotor function reduce balance control during walking resulting in increased fall risk [18–20]. As a result, interventions able to improve walking balance and thus, decrease fall risk in older adults are necessary [13]. An approach to decrease fall risk in older adults is to strengthen their capability to execute self-stable walking patterns [21–23]. The basic premise behind training walking patterns is to allow older adults to walk as planned in the presence of small instabilities. Based on this theoretical framework, walking corresponds to a behavioral attractor, where attractor dynamics are responsible for walking stability [24]. As such, stable walking is based on the passive dynamics of the musculoskeletal system to facilitate foot placement during gait cycles. *In silico* simulation and physical biped-legged models, corroborated with human walking experiments, showed that the mechanisms that underlie foot placement mechanics rely on the passive dynamics of the musculoskeletal system and on the active control from the central nervous system [25–33].

During walking, passive dynamics arises from the biomechanical properties of the body and its mechanical interaction with the environment. Practically, passive dynamics governs walking stability in the fore-aft direction [24, 26, 34–36]. Nevertheless, active control from the central nervous system governs walking stability in the lateral direction [24–26]. Step width variability, expressed as the standard deviation of the mediolateral distance between sequential left and right heel-strikes at double support, reflects the amount of active control from the central nervous system in the frontal plane through lateral foot placement [26]. A recent systematic review and meta-analysis showed that older adults have higher step width variability than younger adults [37]. In older adults, active control is subject to subclinical declines in sensorimotor functions, resulting in increased step width variability [27, 38–42]. Evidence has surfaced to support the link between increased step width variability and high fall risk in older adults [43]. Moreover, step width variability predicted fall incidence among older adults [44].

### Frontal plane gait stability training

Therefore, an intervention to improve walking stability in older adults (and decrease fall risk, thereafter) would be more effective if it targeted to decrease the excessive amount of step width variability. Currently, external stabilization devices and body weight support systems can be used to offload the need of active control and decrease the amount of step width variability during walking [27, 45–48]. This has implications for walking stability intervention in older adults, which could be directed to exploit the mechanical features of gait dynamics, such as motion-dependent torques [49]. Previous studies showed that passive dynamics are less sensitive to age-related deficiencies of active control or the lack thereof [26, 31, 32]. For example, it was postulated that the ability to gradually offload the need of active control in treadmill walking through external devices can be used in rehabilitation medicine for walking stability in older adults [24, 45]. However, step width variability can be decreased by increasing older adults’ ability to control foot placement as well [47].

Recently, it has been found that active control during walking in any direction is dependent on the direction of progression [50, 51]. Specifically, when performing sideways walking, where the mediolateral direction is the direction of progression instead of the anteroposterior, the participants experienced a reversal of what is found in typical forward walking; the mediolateral direction had a lower amount of variability than the anteroposterior direction [51]. Practically, when walking laterally, side stepping became the primary direction of progression, and step width variability was less than step length variability. This implies that all planes of motion can benefit from both active and passive control properties. Exercise-based interventions attempting to improve walking stability and reduce fall risk, would not need to always target the mediolateral direction during typical walking. Therefore, we suggest that adaptations from sideways walking training could transfer to improve gait stability during forward walking. This suggested transfer effect, could provide an alternative intervention approach to reduce fall risk in older adults.

The objectives of this study were to (i) determine the feasibility of implementing a novel 6-week sideways walking exercise intervention for older adults and (ii) to collect preliminary evidence of efficacy of such intervention on risk-of-falling related outcomes. The specific feasibility objectives of the study were (i) to determine the eligibility criteria, (ii) to evaluate the recruitment capability and the characteristics of the sample who expressed interest to participate in the study, (iii) to evaluate the fidelity with which the intervention was implemented in terms of compliance with the protocol and adherence to the procedure (i.e., participant and instructor fidelity), and (iv) to evaluate the feasibility and suitability of data collection procedures and the risk-of-falling related outcomes measures [52]. Furthermore, it was hypothesized that the intervention would improve risk-of-falling related outcomes. It was also expected that the intervention will result in a decrease in the amount of step width variability and an increase in walking speed during forward walking at post-intervention. Moreover, the effects would be retained for 6 weeks after the completion of the intervention.

## METHODS

### Design

We completed a 6-week, single-arm pilot study of a sideways walking intervention with baseline, post-intervention, and retention measurements of risk-of-falling related outcomes. Reporting followed the Consolidated Standards of Reporting Trials (CONSORT) statements for randomized pilot and feasibility trials [53]. To ensure completeness of reporting, and replication of the intervention we followed the Template for Intervention Description and Replication (TiDieR) guidelines [54], which is recommended as extension on the CONSORT guidelines [53].

### Participants

Fifteen older adults enrolled in the study. Inclusion criteria were (i) ≥ 65 years, (ii) be independently residing in the community, and (iii) ability to walk independently without walking aid and without the help of another person. Participants were not eligible if they (i) had a neurological disorder or progressive neurologic condition, (ii) had a musculoskeletal disorder or injury that could affect gait, (iii) had a surgery within the past 6 months, (iv) had a history of a cardiovascular event, and (v) were participating in any other studies that involves walking, balance, or exercise intervention.

Participants were recruited from 3 sources: (i) local retirement community, (ii) employees of the University of Nebraska at Omaha, and (iii) a sample of 190 older adults from the Mind & Brain Health Labs (MBHL) registry of the University of Nebraska Medical Center (UNMC). The MBHL registry provided records of older adults who met our inclusion/exclusion criteria. Between September 2017 and March 2018, one of the authors (AS) went to local retirement homes, fitness classes, and libraries to talk about the research and to distribute approved flyers. Moreover, an invitation to participate to the study was sent by email to all of the 190 members of the MBHL registry. A notice seeking volunteers was also announced to the university’s employees through campus-wide email posts linked to text on university’s website news page. Interested older adults contacted us by email or telephone and a screening visit at the Biomechanics Research Building was scheduled. Eligible older adults were identified by one of the authors (AS), and they asked whether they would like to participate in the study. All participants were asked to read, understand, and sign an informed consent form approved by the Institutional Review Board of the UNMC prior to participating in the study.

### Sideways walking intervention

Before the first session, the participants were given a visual demonstration of sideways walking. Precise instructions were as such: (i) ‘keep the head up while stepping laterally’, (ii) ‘do not cross feet at any point’, (iii) ‘feet and legs are to be pointed in the same direction as the body’, and (iv) ‘at no point can both feet be off the ground’. Every session began with ‘warm-up’ exercises, which included 200–300 m forward walking at self-selected speed and stretching exercises at the comfort level of the participants. After the ‘warm-up,’ the participants started the sideways walking training. The 6-week intervention was performed at the indoor walking track (circumference of about 200 m) of the Recreation Building of the University of Nebraska at Omaha. The 10 m walkway was located at the straight part of the track. We used masking tape of distinctive color to create starting and finish lines on the track. All training sessions had a single participant and were supervised by one of the authors (AS).

The load of the training was based on the American College of Sports Medicine guidelines for older adults [55], which recommend 20–30 min on 2–3 days∙week^− 1^ for neuromotor exercises (balance, agility, coordination and gait). Thus, each participant was trained 3 days∙week^− 1^ for 6 weeks, resulting in a total of 18 sessions. Each participant performed 6 trials∙session^− 1^ that were alternated with a rest period of 1–3 min [55, 56]. The participants were informed that they could choose their rest time but should be at least 1 min but no more than 3 min. Each trial consisted of 3 min sideways walking across a 10 m walkway changing body direction at the ends, thus alternating lead and lag limbs. Each training session lasted 30–45 min. The first session was at the participant’s self-selected sideways walking pace. The participants were given instructions that they should strive to increase their pace if they can, as they progress through the 18 sessions. The participants were informed that they could increase their pace at the start of each trial but may not decrease it at the next session. The time to cover the 10 m sections in each trial was manually recorded. Time stopped when the leading foot crossed the finish line. The averaged recorded time at each session was used to check participants’ adherence to the intervention protocol – i.e., whether recorded time was decreased at each consecutive training session. The session was rescheduled when a participant reported a level of muscle soreness or joint pain that prevented them for maintaining the previous walking speed. When this became evident after a session had already commenced, the session was curtailed but not rescheduled. For safety reasons, sideways walking was performed next to a horizontal handrail to grasp if required. A staff member helping AS was standing next to the participant to record walking time, monitor rest time, and to help as an added safety measure.

### Feasibility outcomes

To evaluate the eligibility criteria, we used the ratio of included participants to those who did not meet the eligibility criteria. Participant and instructor fidelity at the intervention protocol was assessed by monitoring the walking pace at all trials per session. The feasibility and suitability of intervention outcomes was assessed by measuring the extent of the missing data, and ceiling or floor effects. Feasibility was measured by the ability to recruit and retain older adults until complete the follow-up (i.e., 6 weeks after the completion of the intervention). The study was considered feasible if we were able to recruit 3 participants∙week^− 1^, and if ≥ 80 % of the sample was able to complete the follow-up.

### Intervention outcomes

Intervention outcomes were walking speed, gait variability (variability of step width, step length, stride time, and stance time), the Timed Up and Go test (TUG), the Berg Balance Scale (BBS), and the Falls Efficacy Scale-International (FES-I). Walking speed is a predictor of fall risk (when is less than 1 m·sec^− 1^), and of disability, mortality, and adverse events in older adults [57–61]. Change in walking speed near 0.05 m·sec^− 1^ is small but meaningful and change near 0.10 m·sec^− 1^ is substantial [62]. Increased variability of spatial (step length and width) and temporal (stride and stance time) gait characteristics compromises gait performance and increase the tendency of older adults to fall [63, 64]. Stance time variability is an indicator of preclinical disability mobility (when stance time variability ≥ 0.034 sec) [65], while step width variability ≥ 2.5 cm is considered excessive [37]. Meaningful changes are 0.25 cm for step length variability, and 0.01 sec for stance time variability [66]. The TUG test was designed for assessing mobility in older adults [67], and has been used for predicting fall risk (when > 12 sec) [57], as well as for screening for frailty in older adults [68]. The BBS (14 items, max score: 56) is a valid and reliable test to measure the functional balance in older adults and predicts fall risk (when < 50 points) [57, 69, 70]. The FES-I is a valid and reliable questionnaire (16 items, max score: 64) to assess confidence in the performance of activities relevant to daily life and can be used to enhance confidence in level of fall risk (when > 24 points) [57, 71, 72]. Additional measurements included: the Mini-Mental State Examination score (MMSE) (12 items, max score: 30) to measure cognitive impairment [73]; the short form of Geriatric Depression Scale (GDS) (15 items, max score: 15) to assess older adults for depression [74]; the short form of Brief Pain Inventory score (BPI) (4 Pain severity items, max score: 40; 7 Pain interference items, max score: 70) to measure the impact of pain on daily functions [75]. Furthermore, participants have been asked if they had sustained 2 or more falls in the past year. A fall was defined as an event that caused participants to rest on the floor.

### Data collection and analysis

Data were collected at the Biomechanics Research Building at baseline, post-intervention, and retention period (6 weeks following completion of the intervention). The building featured a 3D motion capture system with 17 high-speed Raptor cameras (Motion Analysis Corporation, Santa Rosa, CA, US) synchronized with an instrumented treadmill (AMTI, Watertown, MA, US). Upon arrival at the Biomechanics Research Building the participants changed into a tight-fitting suit. Then the participants walked for 3 min on a treadmill at a self-chosen pace to ‘warm-up’.

####  Baseline evaluation

After the warming up, the participants performed the TUG and BBS, completed the clinical questionnaires (FES-I, BPI, GDS, and MMSE), and were asked for previous falls. For the walking speed test, the participants asked to self-select a treadmill walking speed based on comfort level. The participants started walking at treadmill’s slowest speed and then incrementally (intervals of 0.17 m∙sec^− 1^) the speed was increasing until the participant stated that this was their preferred comfortable speed. The treadmill was then increased another increment, so that the participant could confirm that the previous speed was the preferred speed. This procedure was continued and repeated until successful confirmation that a comfortable speed was reached. Then, retroreflective markers were placed at anatomical locations to gather kinematics data during walking on the treadmill at sampling rate of 100 Hz. The participants performed 3 trials of 3 min walking on a treadmill at their preferred comfortable speed, alternated with 2 min of rest. Participants wore a harness during all treadmill trials.

#### Post‐interventionn evaluation

Following the 6-week sideways walking intervention, the participants performed a post-intervention assessment. The data collection mirrored the baseline assessment with additional trials on treadmill to enable possible comparisons to be made with speed both fixed across sessions (at baseline preferred speed) and free to reflect functional post-intervention differences (post-intervention session using newly determined self-selected preferred speed). Participants performed 6 trials of 3 min treadmill walking. Three trials were at the baseline speed and the other 3 trials were at the post-intervention preferred speed. When there was not post-intervention difference on the preferred speed, the participants performed only 3 trials at the baseline speed. Participants returned 6 weeks after the completion of the intervention for the retention assessment. The data collection at the retention mirrored the post-intervention assessment. Additionally, one of the authors (AS) interviewed the participants whether they continued sideways walking training independently at home following the training period. The answers and comments of the participants were recorded.

We determined gait events from the filtered (low-pass Butterworth, 6 Hz cut-off frequency) heel and toe markers trajectories using custom MATLAB code (v. R2019a, The MathWorks, Natick, MA, US). We used the standard deviation of step width (mediolateral distance between the locations of the sequential left and right heel strikes), step length (anteroposterior distance between the locations of the sequential left and right heel strikes), stride time (the time between 2 consecutive ipsilateral heel strikes), and stance time (the time from heel strike to toe off of the same foot) to evaluate gait variability. Previous studies suggested that within-subject standard deviation of step width is more suitable to express step width variability as the coefficient of variation is applicable only to ratio data, and the step width is considered interval data as it is not bounded by a meaningful zero point [37, 76]. Gait parameters were analyzed from both legs.

### Sample size

A convenience sample size of 15 participants was included. The sample size was based on feasibility. However, with 80 % power and a two-tailed alpha error of 5 % and with an estimate walking speed change of 0.1 ± 0.125 m·sec^− 1^ (i.e., meaningful change [62]) this sample size could detect an effect size of ES = 0.80 (Cohen’s d statistic). The sample size was calculated by using the statistical program G*Power [77].

### Statistical methods

The outcomes measures of walking speed, TUG, and gait variability (step width variability, step length variability, stance variability, and stride time variability) were analyzed separately using a repeated measures ANOVA with *Time* (baseline, post-intervention, and retention assessments) as within factor. When sphericity was violated, a Greenhouse-Geisser correction was applied. The average values at each *Time* level were computed for the statistical analysis. If the ANOVA revealed effects (*p* < 0.05), further univariate comparisons were performed using a planned (simple) contrast in which all conditions were compared with the baseline. For the ANOVA comparisons, the Cohen’s *f* effect size was reported (*f* < 0.10 negligible, *f* < 0.25 small, *f* < 0.40 moderate, otherwise large effect). Responsiveness of outcomes was reported using the ES statistic, this is the mean change between baseline and post-intervention divided by the standard deviation of the measurement at baseline [78, 79] (| ES | < 0.20 negligible, | ES | < 0.50 small and | ES | < 0.80 moderate, otherwise large effect). The ordinal outcomes of the clinical tests (BBS, FES-I) were analyzed separately using Friedman test, followed if needed by Wilcoxon singed-rank test. Kendall’s *W* coefficient of concordance was used to report effect size (*W* < 0.10 negligible, *W* < 0.25 small, *W* < 0.40 moderate, otherwise large effect). Significance level was set at α = 0.05. Statistical analyses were performed in R software Version 3.6.3 (R Foundation for statistical computing, Vienna, Austria) [80] using the *afex* [81], *emmeans* [82], *sjstats* [83], and *rstatix* [84] packages.

## Results

**Fig. 1:**
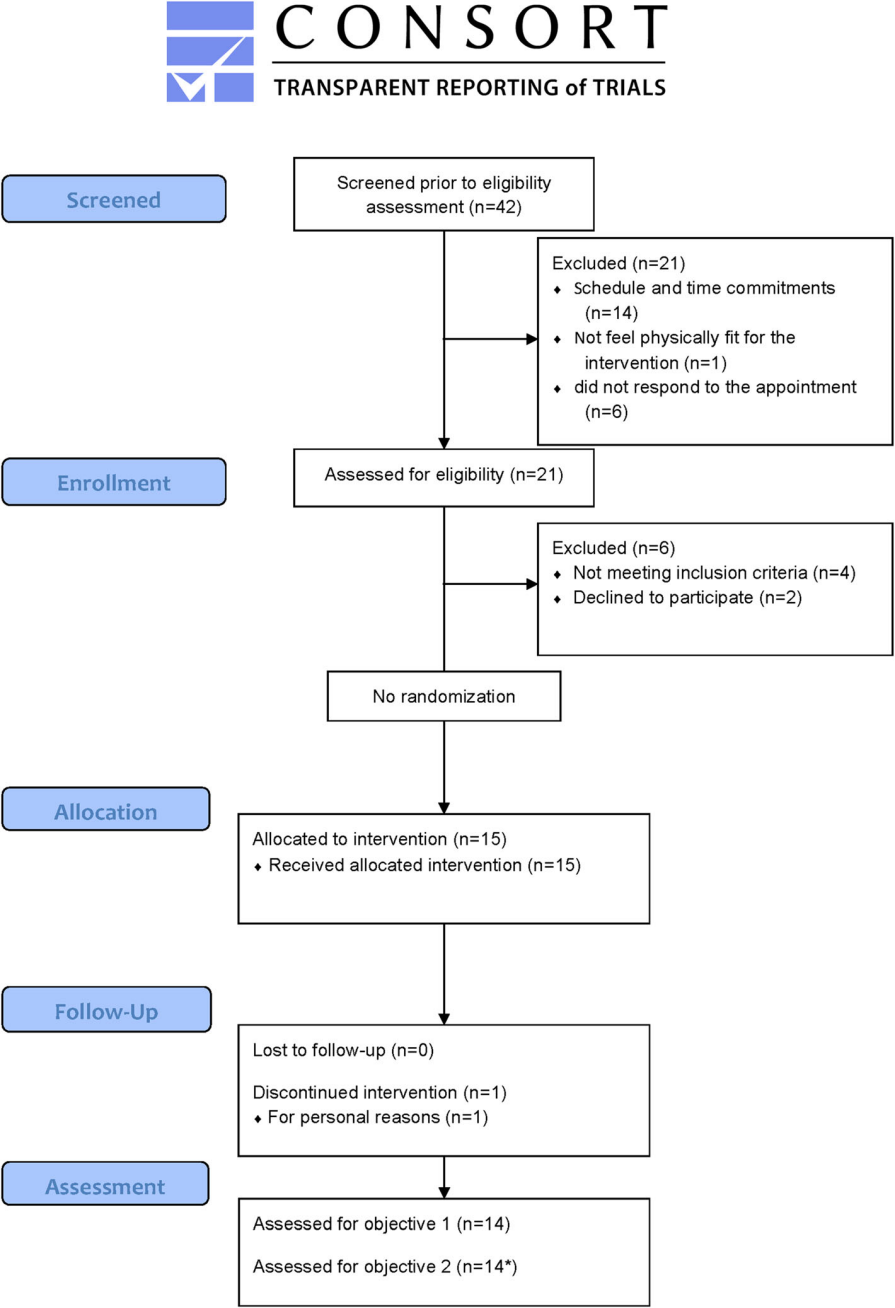
Participant recruitment and study enrolment flow chart (*for treadmill walking speed and gait variability outcomes, *n* = 12)

The flow of participants through the study is shown in Fig. 1. The demographics and baseline characteristics of the 14 participants who completed the 6 weeks intervention are shown in Table 1.

**Table 1:** Demographics and baseline characteristics of the 14 participants who completed the sideways walking intervention

	Older adults (*n* = 14)
Age ^a^	70 ± 4 years (Males 68 ± 1 years; Females: 70 ± 5 years)
Height ^a^	164 ± 10 cm (Males 178 ± 8 cm; Females: 160 ± 8 cm)
Mass ^a^	73 ± 16 kg (Males 74 ± 1 kg; Females: 73 ± 18 kg)
Body mass index ^a^	27 ± 6 Kg/m^2^
Ethnicity	1 African American; 13 White
Sex (Females-to-males)	11:3
Non-fallers to fallers	10:4
GDS ^b^	0.00 ± 0.75
MMSE ^b^	30.0 ± 0.75
BPI: Pain severity ^b^	0.25 ± 0.69
BPI: Pain interference ^b^	0.00 ± 0.10

^a^ values are mean ± standard deviation; ^b^ values are median ± interquartile range; Abbreviations: *BPI* Brief Pain Inventory; *GDS* Geriatric Depression Scale; *MMSE* Mini-Mental State Examination

### Recruitment and retention capability

**Fig. 2:**
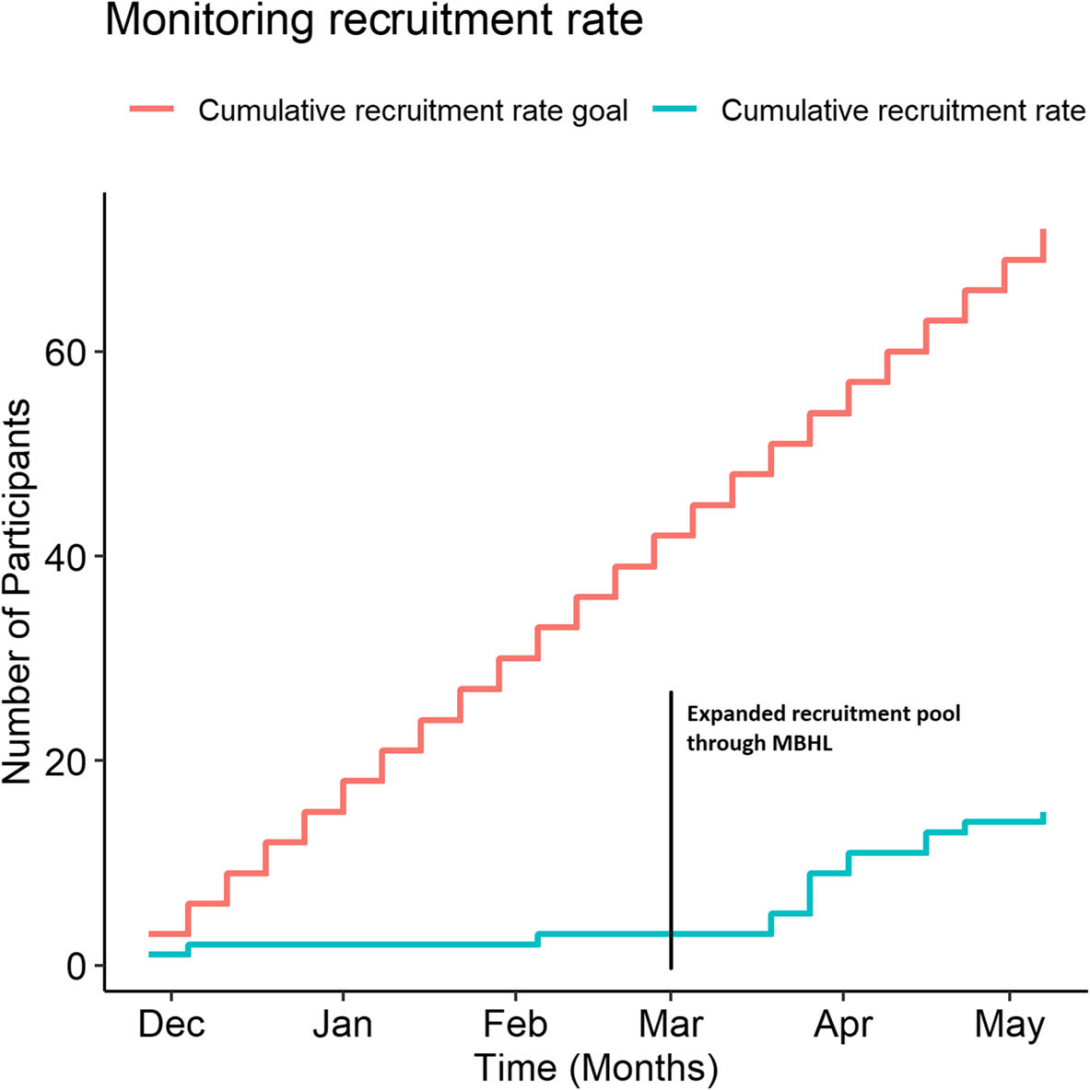
Progress of participant recruitment rate compared to study’s goal rate

Enrollment started the second half of November 2017 and was completed in May 2018 (Fig. 2). Starting from March 2017, we were able to pool participants also from the MBHL registry enabling us to achieve study’s goal rate (3 participants∙week^− 1^). In total, forty-two individuals expressed interest in participating in the study, 21 of whom were unable to enroll because of schedule and time commitments, felt not physically fit, or did not respond to the appointment. Twenty-one individuals were assessed for eligibility, 4 of whom were excluded because they did not meet the eligibility criteria and 2 more because refused to participate without a stipend. Therefore, 15 participants were enrolled (11 participants from the MBHL registry). One participant decided not to continue after 4 weeks of training due to personal reasons (7 % attrition rate). There was no loss regarding the follow-up (100 % follow-up).

### Fidelity of intervention delivery

**Fig. 3:**
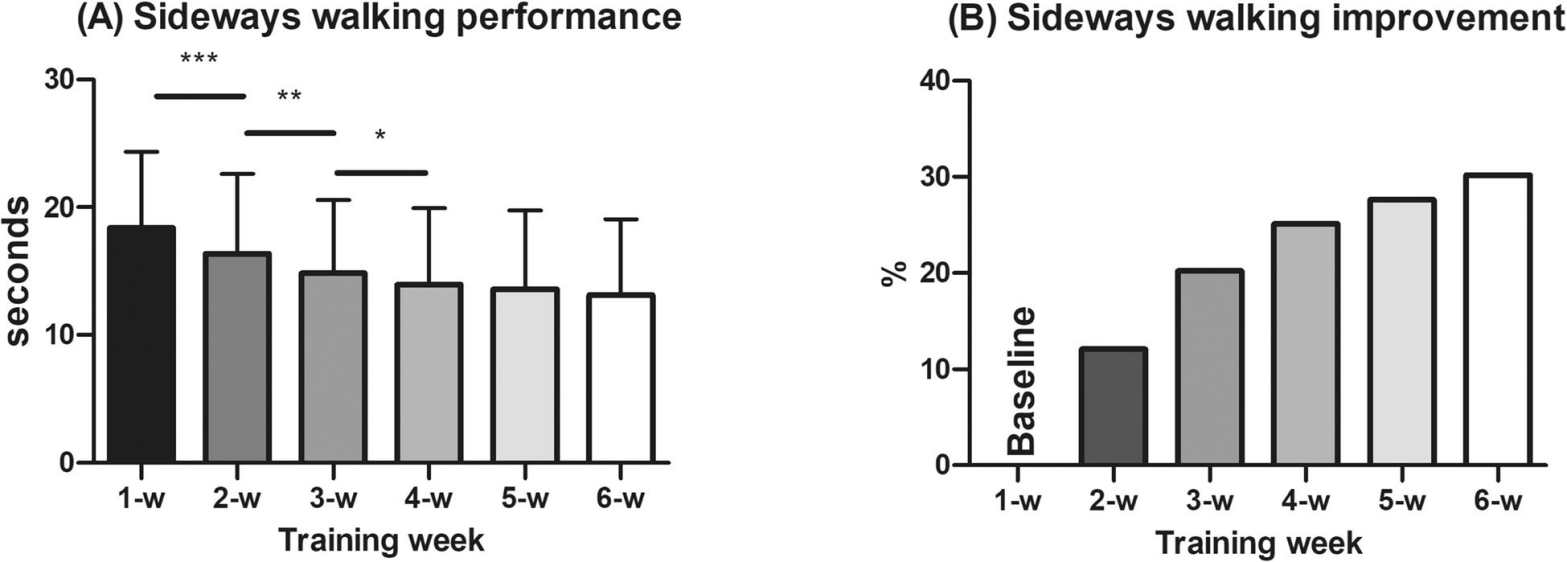
**a** Mean weekly changes on performance and **b** average improvement over the 6-week sideways walking intervention. Performance was measured as the time in seconds needed to cover 10 m walking sideways during the trials (* *p* < 0.05; ** *p* < 0.01; *** *p* < 0.001)

The results showed that older adults followed the instructions and complete the training as required by the protocol. They got faster week by week until they reached a threshold in their sideways walking pace on the 4th week of intervention (Fig. 3a). On average, at the 4th week of intervention the older adults were walking sideways 25 % faster as compared to baseline (Fig. 3b). The increase in sideways walking speed from 4th to 6th week was 5 %.

### Safety

No serious adverse events were recorded during the intervention. Occasionally, minor complaints relating to stiffness, muscle soreness, or dizziness (n = 3) were reported. Participants did not report any fall at any point during the trial and follow-up periods.

### Intervention outcomes

The scores of cognitive impairment (when MMSE < 25 points) [57], depression (when GDS < 6 points), [57] and pain (when BPI: pain interference score < 4.7 points, or BPI: pain severity score < 5.6 points) tests indicate that fall risk at baseline could be considered low [85] (Table 1). The outcome measures of the participants who completed the 6-week intervention are shown in Table 2. Nine participants improved their walking speed, and 3 participants maintained the baseline walking speed at post-intervention. TUG was consistent with low fall risk, and FES-I indicated that they had low fear of falling. BBS showed ceiling effects (7 participants obtained the highest possible score; 12 participants clustered at the highest 10 % of possible score), while FES-I showed floor effects (3 participants obtained the lowest possible score; 10 participants clustered at the lowest 10 % of possible score) at baseline.

**Table 2:** Outcome measures at each assessment and responsiveness

Variable	Intervention effect					Responsiveness					
	Baseline	Post	Retention			Cont. Baseline-Post			Cont. Baseline-Retention		
	Mean ± SD	Mean ± SD	Mean ± SD	*p-*value	EF	%Δ	*p-*value	ES	%Δ	*p-*value	ES
Speed (m·sec^− 1^) ^a, c^	1.11 ± 0.20	1.27 ± 0.21	1.32 ± 0.25	0.001	1.16	15.7	< 0 0.001	0.82	19.3	< 0 0.001	1.03
TUG (sec) ^a^	10.16 ± 1.51	8.79 ± 1.72	8.72 ± 0.98	< 0.001	0.95	−13.6	< 0.001	−0.90	−13.2	< 0.001	−0.95
FES-I ^b^	19.00 ± 5.00	19.00 ± 4.00	17.00 ± 3.00	0.009	0.37	0.0	0.565	−0.11	−5.7	0.016	−0.56
BBS ^b^	55.50 ± 1.75	56.00 ± 0.75	56.00 ± 1.00	0.580	0.04	0.0	0.673	0.29	0.0	0.357	0.29
Stance time variability (sec) ^a, c^	0.018 ± 0.006	0.015 ± 0.004	0.014 ± 0.005	0.026	0.73	−18.0	0.009	−0.58	−18.6	0.006	−0.73
Step width variability (cm) ^a, c^	2.30 ± 0.47	2.15 ± 0.36	2.12 ± 0.38	0.045	0.57	−5.7	0.048	−0.33	−6.8	0.021	−0.39
Step length variability (cm) ^a, c^	1.80 ± 0.43	1.60 ± 0.47	1.56 ± 0.57	0.099	0.52	−10.8	0.074	−0.48	−12.9	0.034	−0.57
Stride time variability (sec) ^a, c^	0.021 ± 0.006	0.018 ± 0.006	0.017 ± 0.007	0.107	0.52	−13.7	0.076	−0.56	−16.0	0.031	−0.69

^a^ values are mean ± standard deviation; ^b^ values are median ± interquartile range; ^c^*n* = 12

Abbreviations: *%Δ* mean or median percentage change from baseline; *Cont.* Contrast; *EF *Cohen’s *f* or Kendall’s *W* index (< 0.10 negligible, < 0.25 small, < 0.40 moderate, otherwise large effect); *ES *effect size index (|ES| < 0.20 negligible, |ES| < 0.50 small and |ES| < 0.80 moderate, otherwise large effect

### Efficacy of intervention

Repeated measures ANOVA revealed that there was an effect of *Time* on walking speed (*F*(1.21, 13.33) = 14.9, *p* = 0.001, *f* = 1.16), step width variability (*F*(2, 22) = 3.59, *p* = 0.045, *f* = 0.57), stance time variability (*F*(1.17, 12.92) = 5.91, *p* = 0.026, *f* = 0.73), and TUG score (*F*(2, 26) = 11.83, *p* < 0.001, *f* = 0.95). Friedman test showed that there was an effect of *Time* on FES-I score (*χ*^2^(2) = 9.5, *p* = 0.009, *W* = 0.37). Planned contrasts revealed that a 6-week sideways walking intervention increased walking speed (*t*(22) = 4.13, *p* < 0.001, ES = 0.82), and decreased step width variability (*t*(22) = − 2.10, *p* = 0.048, ES = − 0.33), stance time variability (*t*(22) = − 2.88, *p* = 0.009, ES = − 0.73), and TUG (*t*(26) = − 4.10, *p* < 0.001, ES = − 0.90) from baseline to post-intervention. These results were retained 6 weeks after the completion of the intervention (walking speed: *t*(22) = 5.16, *p* < 0.001, ES = 1.03; step width variability: *t*(22) = − 2.49, *p* = 0.021, ES = − 0.39; stance time variability: *t*(22) = − 3.07, *p* = 0.006, ES = − 0.73; TUG score: *t*(26) = − 4.31, *p* < 0.001, ES = − 0.95). Wilcoxon signed-rank test revealed that FES-I decreased from post-intervention to 6 weeks after the completion of the intervention (*Z* = 43, *p* = 0.016, ES = − 0.56).

## Discussion

In this study of community-dwelling older adults we demonstrated the feasibility of the 6-week sideways walking intervention and preliminary evidence in favor of the efficacy of the intervention in reducing certain risk-of-falling related outcomes.

### Feasibility of intervention

Overall, the protocol was robust, and the intervention was safe and acceptable from the participants. Low study uptake and poor recruitment rate were the main limiting factors. Many potential participants were reluctant to commit to a 6-week intervention and that might have affected sample characteristics. Recruitment rate improved by expanding the recruitment pool through MBHL. Therefore, the inclusion of community partners to assist with recruitment should be considered. Educational materials stating clear personal benefits gained from participation could be used to promote the study in community organizations and encourage participation. The fact that recruitment during the winter season was unsuccessful indicates that timing of conducting recruitment and implementing intervention should be considered as well [86, 87]. Previous research showed that weather conditions, such as cold, influences older adults’ attendance and adherence to exercise classes [88]. Of course, such effects could not be generalized to all parts of the US as winters in Nebraska, where this study took place, could be more severe than other locations.

The presence of floor effects on FES-I and ceiling effects on BBS at baseline suggests that healthy functioning older adults were engaged in the study [89]. Thus, the use of alternative clinical tools should also be considered. The Fullerton Advanced Balance test could be used instead of BBS as it was designed to measure functional balance in older adults [90, 91]. In our study, fear of falling was assessed using the FES-I. Incorporating the modified Gait Efficacy Scale [92], instead of the FES-I, may be more appropriate to assess fear in walking-related activities for community-dwelling older adults. Additionally, future studies could target older adults at high risk for falls (e.g., fallers older adults). It would be interesting to see whether a sideways walking intervention would be able to prevent falls in this population. Moreover, it would be interesting to investigate whether older adults with frontal plane gait instability would benefit more from a targeted intervention of sideways walking that is able to decrease step width variability than other type of gait training.

### Qualitative results related to the intervention

The 6 weeks sideways walking intervention was broadly acceptable to the participants. They were motivated to participate, and they were often trying to exercise at home. Five participants practiced sideways walking at home or in community. Moreover, the participant who dropped out did not cite motivational reasons. Some of the comments that were received were as follows:

I have retired a few months ago and this consistent attendance on the program makes me feel good…with energy”; “I am doing it at home, it is so funny, everyone is watching me walking as crub! It is so funny”; “It reminds me the ballet classes when I was young!

Sideways walking is a simple, natural movement, and is a minimal-cost accessible solution that could be translated easily into the real world. It requires minimal available resources. No training specialists, equipment, or facilities are needed. Moreover, older adults can begin the intervention on their own and without any particular preparation, either indoors or outdoors.

### Efficacy of sideways walking intervention

We were able to confirm the hypothesis that sideways walking would improve risk-of-falling related outcomes, and the effects would be retained for 6 weeks after the completion of the intervention. Improvements were noted for walking speed, TUG, stance time variability, step width variability, and FES-I (*p* < 0.05). Specifically, walking speed was more sensitive to the impact of sideways walking intervention than were the other outcomes. Large ES were seen at post-intervention and 6 weeks after the completion of the intervention. These large ES were equated with substantial clinically gains in older adults’ gait performance (> 0.15 m·sec^− 1^) [62]. Walking speed at preferred pace is an important phenotypic marker of health and functional status [93]. Walking speed > 1.2 m·sec^− 1^ was found to associate with healthier aging and exceptional life expectancy [58, 61].

TUG was sensitive to the impact of sideways walking intervention. The large ES that were seen at post-intervention and at follow-up indicated substantial change over time (about 13 % from baseline). Previous studies indicated that for claiming a ‘real’ effect over a period of 4 weeks, TUG needs to change by more than 15 % from baseline in older adults [94], and by more than 10.18 % in a population aged 30–74 years [95]. TUG score at baseline (10.16 ± 1.51 sec) was at the upper end of the range of previously reported values for community-dwelling older adults (9.2 sec; CI95 % = 8.2–10.2) [96]. TUG score at baseline was also greater than the 9 sec cut-off value for future incidence of disability [97]. Moreover, TUG more than 10 sec is associated with increased risk of all-cause mortality [98]. Our intervention reduced TUG to the lower end of the reported range (8.79 ± 1.72 sec) [96]. Lower TUG scores reduce the risk of all-cause mortality. A recent epidemiological study in older adults (*N* = 864; deaths = 428) reported a significant association (hazard ratio [HR]: 1.28; CI95 % = 0.96–1.71) between TUG and all-cause mortality for those who were fastest (8.4 ± 1.2 sec) compared with those who were slower (10.5 ± 0.5 sec) [99].

Stance time variability at baseline was less than the 0.034 sec cut-off value for future mobility disability [65]. Sideways walking intervention decreased stance time variability by about 18 % from baseline. This was a moderate change in terms of ES. However, it was not equated with a clinically meaningful change [66]. Short-term and long-term gains in step width variability were substantial in terms of ES [66]. In a recently conducted meta-analysis, it was verified that step width variability is higher in older adults than in young adults [37]. Moreover, it was identified that step width variability values above 2.50 cm are excessive, while values lesser than 1.97 cm are within the normative range [37]. Our intervention was able to lower step width variability in our older adults from an average of 2.30 cm to 2.15 cm, while at follow up was at 2.12 cm. This is a preliminary evidence that sideways walking intervention can reduce the requirements of frontal plane active control in older adults’ gait during forward walking. Sideways walking had a moderate effect on stride time variability and step length variability. Nevertheless, long-term gains in step length variability were substantial in terms of ES (0.24 cm), and close to a clinically meaningful change (≥ 0.25 cm) [66].

FES-I scores at baseline indicated that participants had relatively low fear of falling [57, 71, 72]. Floor effects seen at baseline may have an impact on the responsiveness of FES-I. However, the results showed a beneficial follow-up effect of sideways walking on FES-I. High BBS scores at baseline supported that participants had good functional balance with low fall risk [57, 69, 70]. The small change in terms of ES at post-intervention indicates that BBS was not responsive to the training, possibly because of the high scores at baseline, which caused ceiling effects for this assessment.

### Limitations

Although simultaneous participation in any other competitive intervention was considered an exclusion criterion, a limitation is that we did not include a washout phase for those older adults who may had completed an exercise intervention just prior to screening. Another limitation is that during the sideways walking intervention, the participants walked with the staff side-by-side. According to a recent study, it could be an interchange of information between older adults and staff that is accomplished through the matching of the fractal properties of stride intervals; the most complex system (staff) may attract the less complex (participants), yielding in an increase of complexity in the older adult that could be reflected on the gait patterns [100]. We do not know how these limitations could affect our intervention. A washout phase should be included before baseline assessment. The staff could monitor the participant walking further away.

### Future research

This pilot study was conducted to support a large-scale randomized controlled trial on the use of sideways walking to decrease risk of falling in older adults.

## Conclusions

We concluded that a 6-week sideways walking is a feasible exercise intervention to improve risk-of-falling related outcomes in this population and settings.

## Data Availability

The datasets analyzed during the current study are available from the corresponding author on reasonable request.

## Abbreviations

BBS: Berg Balance Scale
BPI: Brief Pain Inventory
CONSORT: Consolidated Standards of Reporting Trials
ES: Effect Size
FES-I: Falls Efficacy Scale-International
GDS: Geriatric Depression Scale
TiDieR: Template for Intervention Description and Replication
TUG: Timed Up and Go
MBHL: Mind & Brain Health Labs
MMSE: Mini-Mental State Examination
UNMC: University of Nebraska Medical Center
